# Spatial Signature Estimation with an Uncalibrated Uniform Linear Array ^†^

**DOI:** 10.3390/s150613899

**Published:** 2015-06-12

**Authors:** Xiang Cao, Jingmin Xin, Yoshifumi Nishio, Nanning Zheng

**Affiliations:** 1 Institute of Artificial Intelligence and Robotics, Xi'an Jiaotong University, Xi'an 710049, China; E-Mails: cao.xiang@stu.xjtu.edu.cn (X.C.); jxin@mail.xjtu.edu.cn (J.X.); nnzheng@mail.xjtu.edu.cn (N.Z.); 2 Department of Electrical and Electronic Engineering, Tokushima University, Tokushima 770-8506, Japan

**Keywords:** array signal processing, auto-calibration, parameter estimation

## Abstract

In this paper, the problem of spatial signature estimation using a uniform linear array (ULA) with unknown sensor gain and phase errors is considered. As is well known, the directions-of-arrival (DOAs) can only be determined within an unknown rotational angle in this array model. However, the phase ambiguity has no impact on the identification of the spatial signature. Two auto-calibration methods are presented for spatial signature estimation. In our methods, the rotational DOAs and model error parameters are firstly obtained, and the spatial signature is subsequently calculated. The first method extracts two subarrays from the ULA to construct an estimator, and the elements of the array can be used several times in one subarray. The other fully exploits multiple invariances in the interior of the sensor array, and a multidimensional nonlinear problem is formulated. A Gauss–Newton iterative algorithm is applied for solving it. The first method can provide excellent initial inputs for the second one. The effectiveness of the proposed algorithms is demonstrated by several simulation results.

## 1. Introduction

In wireless communication, a multiple antenna array at base stations is usually used for combating fading, suppressing co-channel interference and increasing capacity [1,2]. If the spatial signatures are exactly known, signals from different users can be separated at the base station array. However, the user spatial signatures are usually unknown in practice and, therefore, must be estimated.

A natural approach to spatial signature estimation makes use of training sequences transmitted by each user. This is a time-consuming method, and the problems of information transmission rate and synchronization are difficult to overcome. As a result, researchers recently have been paying attention to blind spatial signature estimation techniques. In [3], a blind approach based on parallel factor (PARAFAC) analysis is proposed for spatial signature estimation. This method resorts to the assumption of the diagonal covariance matrix of the signals (*i.e.*, uncorrelated signals) to formulate the PARAFAC model. The time-varying user power loading is exploited to identify this model. The strict requirements for signal and user power can limit practical applications of this method.

Unlike the technique proposed in [3], the most common one utilizes the parametric model of spatial signature. This method is involved in determining the directions-of-arrival (DOAs) of incident signals. For an ideal array output model, many high-resolution methods, such as multiple signal classification (MUSIC) [4] and estimation of signal parameters via rotational invariance techniques (ESPRIT) [5], can achieve high estimating accuracy [6]. However, sensor perturbations (e.g., mutual coupling, gain/phase errors and sensor position uncertainty) are not easily eliminated or compensated [7]. The performance of the subspace-based methods will be severely degraded by these model errors [8,9], and we know from [10–12] that model errors also affect the Cramér–Rao lower bound. Therefore, sensor perturbations must be taken into account for renewing the capability of these approaches. In other words, once the incident signal DOAs and the unknown model errors are obtained, the problem of spatial signature or channel estimation is solved.

Two parametric errors (*i.e.*, statistical and deterministic) [13,14] are usually used to extend the ideal array model. This paper only focuses on the DOA-based spatial signature estimation method under deterministic unknown gain and phase errors. The term deterministic here implies that the unknown error parameter at each element of the array is a complex stable constant during the period of observation.

A way to compensate for the unknown model errors is to make use of signal sources in known directions. The methods for calibrating sensor gain and phase errors are proposed in [15,16]. A combined calibration of gain, phase and mutual coupling, as well as sensor position perturbations using the maximum likelihood approach has been studied in [7]. All of the above calibration methods require knowledge of exact source localizations or the covariance matrix of the ideal array response. This is a practical and valid approach when the model errors keep constant with time. Meanwhile, several so-called auto-calibration approaches have also been developed in the literature. Here, the auto-calibration indicates that array calibration can be accomplished without employing any dummy elements or transmitters at known directions. When the first and second order statistic model of sensor errors is given, an optimal maximum *a posteriori* (MAP) estimator is presented [14,17,18]. The problem of DOA estimation is taken apart from optimization function, and alternative iteration is avoided. Despite such techniques based on MAP being called auto-calibration methods, they need the prior distribution of array perturbation parameters.

In [19], an iterative eigenstructure-based technique of estimating DOAs in the presence of unknown gain and phase errors is presented, and this method can apply to arbitrary array geometries, except a uniform linear array (ULA). For the ULA, a phase ambiguity exists between the diagonal error matrix and the ideal array steering matrix (see [20–22]). Thus, it is impossible to estimate DOA and gain and phase errors simultaneously. A Hermitian Toeplitz structure of the ideal ULA output covariance matrix is exploited in [23,24]. This method takes advantage of the elements' equivalence at every diagonal line of covariance matrix to form two equations, and the sensor gain and phase error vectors can be separately obtained from these two equations. The main drawback of the approach is that the Toeplitz matrix assumption is only established under infinite sampling conditions. This means that if the number of snapshots is fixed, the performance of the algorithm does not improve after increasing the signal-to-noise ratio (SNR) to a certain point.

In addition, ESPRIT can also be extended to this case. The idea firstly emerged in [15] and is applied for estimating spatial signatures in [22,25]. In [22], a closed-form ESPRIT-like method is proposed, and the maximum overlapping two subarrays are selected from the array to construct an estimator. This method is also used for DOA estimation with a partly-calibrated ULA [26]. However, it has been pointed out that any two subarrays' configuration may be inherently suboptimal [27]. This provides the motivation for the estimation strategy that fully exploits the multiple invariances in the array. In fact, a multiple invariance (MI) ESPRIT has been proposed for DOA estimation under an ideal array output model [28]. Because model errors exist, the MI-ESPRIT method will fail in our problem.

In this paper, we consider the problem of spatial signature estimation with the ULA in the presence of unknown gain and phase errors. As is well known, the incident signal DOAs can only be determined within an arbitrary rotation angle in this case. However, this rotational ambiguity has no impact on the identification of spatial signature [22]. The sensor gain and phase errors are different from mutual coupling among elements of the array, and it cannot be affected by other sensors. In addition, the ideal steering matrix of the ULA has a Vandermonde structure. The two properties provide the possibility of selecting subarrays from the uncalibrated array.

In practice, two eigenstructure-based algorithms are presented in this paper. The first one extracts two subarrays from the ULA to construct an estimator, and a closed-form solution is obtained. The difference from the ESPRIT-like method is that one element of the ULA can be used several times in one subarray. The second method fully exploits the multiple invariances in the ULA. It is natural to expect that an estimator formulated by multiple invariances in the array performs better than the one involving only one invariance (e.g., the ESPRIT-like method). In this method, a multidimensional procedure of minimizing a cost function is presented. The Gauss–Newton algorithm [29] is suggested to solve the multidimensional search problem. The choice of the initial values is essential for the global convergence of the iterative method. Fortunately, the excellent initial inputs can be provided by our first method. Our algorithms firstly estimate the rotational DOAs (not absolute DOAs) and error parameters, then obtain the estimation of the spatial signature. The effectiveness of the proposed algorithms is compared with the ESPRIT-like method [22] by several computer simulations. Furthermore, unlike the method proposed in [3], the subsequent use of these two methods, as done here, does not depend on the assumption of uncorrelated incident signals and time-varying user power.

## 2. Data Model

A uniform linear array with *M* sensors receiving narrowband signals from *p* far-field sources and the array output vector ***y*** ∈ 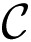
*^M^*^×1^ at time *t* can be expressed as:

(1) y(t)=Γ(γ)A(θ)s(t)+n(t)

where ***s***(*t*) ∈ 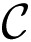
*^p^*^×1^ is the vector of incident signals at time *t*, ***n***(*t*) ∈ 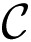
*^M^*^×1^ is the vector of additive noises, the steering matrix ***A***(**θ**) = [ ***a***(θ_1_) ***a***(θ_2_) ⋯ ***a***(θ*_p_*) ] is the ideal array response and:

(2) $$\text{a}(\theta )={\left[\begin{array}{llll} 1 & e^{j\frac{2\pi }{\lambda }d\sin(\theta )} & \cdots & e^{j\frac{2\pi }{\lambda }(M-1)d\sin(\theta )} \end{array}\right]}^{T}$$

Here, operator (·)*^T^* stands for transpose, θ_1_, θ_2_, ⋯ , θ*_p_* are the directions-of-arrival of incident signals and *d* and λ represent the distance between two consecutive sensors and the identical wavelength for all signals, respectively The diagonal matrix **Γ**(*γ*) is given by:

(3) $$\Gamma (\gamma )=\left[\begin{array}{llll} \gamma_{1} & & & \\ & \gamma_{2} & & \\ & & \ddots & \\ & & & \gamma_{M} \end{array}\right]$$

and *γ_i_*, *i* = 1, ⋯ , *M* denotes the deterministic unknown gain and phase error of sensor *i*. The vector *γ* = [*γ*_1_,·⋯ , *γ_M_*]*^T^* is constructed out of the diagonal elements of matrix **Γ**(*γ*). For notational convenience, we omit the arguments of **Γ**(*γ*) and ***A***(**θ**) sometimes.

Two assumptions need to be made. Firstly, signal ***s***(*t*) is a temporally-complex white Gaussian random vector with mean zero, and its covariance matrix ***R_SS_*** has full rank *p*. Secondly, noise ***n***(*t*) is a temporally and spatially complex white Gaussian random vector with mean zero and uncorrelated with incident signals. Then, the so-called signal subspace ***E****_s_* and noise subspace ***E****_n_* can be easily obtained from the eigenvalue decomposition of array output covariance matrix:

(4) $$\text{R}=E\{\text{y}(t){\text{y}}^{H}(t)\}=\sum_{i=1}^{M}\lambda_{i}e_{i}e_{i}^{H}$$

where *E*{·} and (·)*^H^* denote the statistical expectation and the complex conjugate transpose, respectively. The eigenvalues are ordered, such as λ_1_ ≥ λ_2_ ≥ ⋯ ≥ λ*_p_* > λ*_p_*_+1_ = ⋯ = λ*_M_*. The corresponding signal subspace ***E****_s_* and noise subspace ***E****_n_* are given by ***E****_s_* = [ ***e***_1_ ⋯ ***e****_p_* ] and ***E****_n_* = [ ***e****_p_*_+1_ ⋯ ***e****_M_* ].

The focus of this paper is the estimation of spatial signature matrix ***V*** = **Γ**(*γ*)***A***(**θ**) from the *N* snapshots of the array output. One ambiguity for this problem can be observed between the unknown signal vector ***s***(*t*) and ***V*** (*i.e.*, $Vs(t)=\alpha V\cdot \left(\frac{1}{\alpha }s(t)\right)$ for an unknown non-zero scaling *α*). A reasonable constraint for solving this scaling ambiguity is to let the first element of diagonal matrix **Γ**(*γ*) be equal to one.

## 3. Estimation Algorithms

Two new subspace-based approaches are presented for estimating spatial signature matrix ***V*** in this section. The Vandermonde structure of the ideal array steering matrix ***A***(**θ**) provides the opportunity for optimally exploiting one invariance or multiple invariances in the ULA, even though the gain and phase errors exist. Since there is a one-to-one relationship between rows in matrix ***V*** and elements of the array, extracting a subarray from the array is equivalent to picking up rows of matrix ***V***. We make use of these particular properties to construct our estimators. Here, we have to point out that the one-to-one relationship in rows of ***V*** and the elements of the array is not held under mutual coupling errors. The reason is that the response of one element is affected by other elements of the array.

### 3.1. A Novel ESPRIT-Like Method

Define a *q* × (*M* − *ε*) selection matrix ***Ĵ*** consisting of zeros and ones, where *q* = *M* − *ε* + Σ(*l_i_* − 1), *l_i_* denotes that the *i*–th element of the array is used *l_i_* times in one subarray, and the distance between the subarrays is *ε* times the element spacing. Only one element at every row is equal to one, and it corresponds to the selected sensor of the array. However, the number one at each column in ***Ĵ*** indicates the number of repetitions of selected element in one subarray.

Then, the spatial signature matrix ***V*** can be partitioned into two subarrays.

(5) $$\text{J}\Gamma \text{A}=\left[\begin{array}{c} \Gamma_{x}{\text{A}}_{x}\\ \Gamma_{y}{\text{A}}_{x}\Phi^{ɛ} \end{array}\right]$$

where:

(6) $$\text{J}=\left[\begin{array}{l} {\hat{J}}_{1}\\ {\hat{J}}_{2} \end{array}\right]=\left[\begin{array}{cc} {\hat{J}}_{q\times (M-ɛ)} & 0_{q\times ɛ}\\ 0_{q\times ɛ} & {\hat{J}}_{q\times (M-ɛ)} \end{array}\right]$$

**Γ***_x_*, **Γ***_y_* and ***A****_x_* can be calculated by the following equations:

(7) $$\begin{array}{lll} \Gamma_{x}=\text{diag}\{{\hat{J}}_{1}\gamma \}, & \Gamma_{y}=\text{diag}\{{\hat{J}}_{2}\gamma \}, & {\text{A}}_{x}={\hat{J}}_{1}\text{A} \end{array}$$

Here, the operator diag{·} denotes forming a diagonal matrix by taking the given vector as the main diagonal. The displacement diagonal matrix **Φ** is given by:

(8) $$\Phi =\left[\begin{array}{llll} E^{J\frac{2\pi }{\lambda }d\sin(\theta_{1})} & & & \\ & E^{J\frac{2\pi }{\lambda }d\sin(\theta_{2})} & & \\ & & \ddots & \\ & & & E^{J\frac{2\pi }{\lambda }d\sin(\theta_{p})} \end{array}\right]$$

Here, we give an example to illustrate how to choose the selection matrix. Consider a ULA with six elements; a possible selection matrix ***Ĵ***_9×5_ can be expressed as:

(9) $${\hat{J}}_{9\times 5}=\left[\begin{array}{lllll} 1 & 0 & 0 & 0 & 0\\ 0 & 1 & 0 & 0 & 0\\ 0 & 0 & 1 & 0 & 0\\ 0 & 1 & 0 & 0 & 0\\ 0 & 0 & 1 & 0 & 0\\ 0 & 0 & 0 & 1 & 0\\ 0 & 0 & 1 & 0 & 0\\ 0 & 0 & 0 & 1 & 0\\ 0 & 0 & 0 & 0 & 1 \end{array}\right]$$

In this example, the third element of the array is used three times, while the second and fourth one are selected twice in one subarray.

The determinant of the matrix **Γ**(*γ*) is given by:

(10) $$\det(\Gamma (\gamma ))=\prod_{i=1}^{M}\gamma_{i}\neq 0$$

then the rank of the matrix ***V*** is *p*, *i.e.*, Rank(**Γ*A***) = *p*. It is clear that the matrix **Γ*A*** spans the same space as the signal subspace ***E****_s_*. The relationship implies that there exists a nonsingular *p* × *p* matrix ***T*** satisfying:

(11) $${\text{JE}}_{s}=\left[\begin{array}{l} {\text{E}}_{x}\\ {\text{E}}_{y} \end{array}\right]=\text{J}\Gamma \text{AT}$$

Combining Equations (5) and (11) leads to:

(12) $$\begin{array}{ll} {\text{E}}_{x}=\Gamma_{x}{\text{A}}_{x}\text{T}, & {\text{E}}_{y}=\Gamma_{y}{\text{A}}_{x}\bar{\Phi }\text{T} \end{array}$$

where diagonal matrix **Φ̄** = **Φ***^ε^*. Eliminating ***A****_x_* in Equation (12) yields to:

(13) $$\bar{\Gamma }(\bar{\gamma }){\text{E}}_{y}={\text{E}}_{x}\Xi$$

where the *q* × *q* diagonal matrix $\bar{\Gamma }(\bar{\gamma })=\Gamma_{y}^{-1}\Gamma_{x}$ and **Ξ** = ***T***^−1^**Φ̄*T***. The vector *γ̄* can be constructed from the diagonal elements of matrix **Γ̄**(*γ̄*).

Similar to the ESPRIT-like method in [22], **Γ̄**(*γ̄*) and **Ξ** can be estimated from the least squares problem as follows:

(14) $$\hat{\bar{\Gamma }},\hat{\Xi }=\underset{\bar{\Gamma },\Xi }{\arg\min}{\left\|\bar{\Gamma }(\bar{\gamma }){\text{E}}_{y}-{\text{E}}_{x}\Xi \right\|}_{F}^{2}$$

where ∥ · ∥*_F_* denotes the Frobenius norm. The solution for **Ξ** is given by:

(15) $$\hat{\Xi }={\left(E_{x}^{H}E_{x}\right)}^{-1}E_{x}^{H}\bar{\Gamma }(\bar{\gamma })E_{y}$$

Substituting Equation (15) back to Equation (14) and after some manipulation, the least squares problem becomes:

(16) $$\hat{\bar{\gamma }}=\underset{\bar{\gamma }}{\arg\min}{\bar{\gamma }}^{H}\left[\prod_{E_{x}}^{⊥}⊙{\left(E_{y}E_{y}^{H}\right)}^{T}\right]\bar{\gamma }$$

where $\prod_{E_{x}}^{⊥}=I-E_{x}{\left(E_{x}^{H}E_{x}\right)}^{-1}E_{x}^{H}$, and ⊙ stands for the element-wise product of two matrices. From the above equation, an estimate of vector *γ̄* may be obtained from the eigenvector associated with the smallest eigenvalue of the matrix $\prod_{E_{x}}^{⊥}⊙{\left(E_{y}E_{y}^{H}\right)}^{T}$. Here, we note that the constraint may be added to matrix **Ξ**, and some discussions about this question can be found in [22].

Consider the example in Equation (9), the two diagonal matrices **Γ***_x_* and **Γ***_y_* can be expressed as:

(17) $$\Gamma_{x}=\text{diag}\{1,\gamma_{2},\gamma_{3},\gamma_{2},\gamma_{3},\gamma_{4},\gamma_{3},\gamma_{4},\gamma_{5}\}$$

and:

(18) $$\Gamma_{y}=\text{diag}\{\gamma_{2},\gamma_{3},\gamma_{4},\gamma_{3},\gamma_{4},\gamma_{5},\gamma_{4},\gamma_{5},\gamma_{6}\}$$

Recall $\bar{\Gamma }(\bar{\gamma })=\Gamma_{y}^{-1}\Gamma_{x}$; we can see that once an estimate of vector *γ̄* is obtained from Equation (16), it is convenient to obtain the elements of vector *γ*. Furthermore, the matrix **Ξ** can be obtained by substituting the matrix **Γ̄**(*γ̄*) reconstructed from $\hat{\bar{\gamma }}$ back to Equation (15).

The proposed algorithm now is briefly outlined below.

1. Estimate the signal subspace ***Ê****_s_* from the eigendecomposition of the sample covariance matrix $\hat{R}=\frac{1}{N}\sum_{n=1}^{N}y(n)y^{H}(n)$, where *N* is the finite number of snapshots.
2. Calculate ***E****_x_* and ***E****_y_* according to Equation (11), then determine an estimate of vector *γ̄* from Equation (16).
3. Calculate the matrix **Ξ** according to Equation (15), then extract the rotational DOA θ̂*_i_*, *i* = 1, ⋯ , *p*.
4. Construct matrix **Γ**(*γ̂*), and ***A***(**θ̂**), then the spatial signature matrix is estimated as ***V****^* = **Γ**(*γ̂*)***A***(**θ̂**).

## 3.2. An MI-ESPRIT-Like Method

Assume that the array comprises *n* identical subarrays of *m* sensors. Obviously, overlapping subarrays make *M* ≤ *mn*. In addition, it has been pointed out that extracting a subarray from the array is equivalent to picking up *m* rows of matrix ***V*** by a *m* × *M* selection matrix ***J****_i_*. The full row rank matrix ***J****_i_* consists of zeros and ones. Only one element at every row is equal to one, and it corresponds to the selected sensor of the array.

Similar to Equation (11), for the *i*-th subarray, we have:

(19) $$J_{i}E_{s}=J_{i}\Gamma (\gamma )A(\theta )T_{1}$$

where ***T***_1_ is a nonsingular *p* × *p* matrix. Considering *n* subarrays, we have:

(20) $$\bar{J}E_{s}=\left[\begin{array}{c} E_{s1}\\ E_{s2}\\ E_{s3}\\ \vdots \\ E_{sn} \end{array}\right]=\left[\begin{array}{l} \Gamma_{1}A_{1}\\ \Gamma_{2}A_{1}\Phi^{{ɛ}_{1}}\\ \Gamma_{3}A_{1}\Phi^{{ɛ}_{2}}\\ \vdots \\ \Gamma_{n}A_{1}\Phi^{{ɛ}_{n-1}} \end{array}\right]T_{1}$$

where *ε_i_d* denotes the distance between the (*i* + 1)–th subarray and the reference subarray; the matrix ***J****¯* is formed by the selection matrix ***J****_i_* and $\bar{J}={\left[\begin{array}{llll} J_{1}^{T} & J_{2}^{T} & \cdots & J_{n}^{T} \end{array}\right]}^{T}$, ***E****_si_*, and **Γ***_i_* and ***A***_1_ can be calculated by the following equations:

(21) $$\begin{array}{lll} E_{si}=J_{i}E_{s}, & \Gamma_{i}=J_{i}\Gamma (\gamma )J_{i}^{T}, & A_{1}=J_{1}A(\theta ) \end{array}$$

Analogous to the MI-ESPRIT method in [28], the unknown parameter vector **μ** can be obtained from the following least squares problem:

(22) $$\Omega (\mu )={\left\|\left[\begin{array}{c} E_{s1}\\ E_{s2}\\ E_{s3}\\ \vdots \\ E_{sn} \end{array}\right]W^{1/2}-\left[\begin{array}{l} \Gamma_{1}A_{1}\\ \Gamma_{2}A_{1}\Phi^{{ɛ}_{1}}\\ \Gamma_{3}A_{1}\Phi^{{ɛ}_{2}}\\ \vdots \\ \Gamma_{n}A_{1}\Phi^{{ɛ}_{n-1}} \end{array}\right]T_{1}\right\|}_{F}^{2}$$

and:

(23) $$\begin{array}{r} \mu =[\begin{array}{ll} {\bar{\gamma }}_{2}\cdots {\bar{\gamma }}_{M} & {\tilde{\gamma }}_{2}\cdots {\tilde{\gamma }}_{M} \end{array}\\ {\begin{array}{ll} \rho_{1}\cdots \rho_{p} & \theta_{1}\cdots \theta_{p} \end{array}]}^{T} \end{array}$$

where ***W*** is a Hermitian positive definite weighting matrix and *γ̄_i_* and *γ͂_i_*, respectively, denote the real part and image part of *γ_i_*. Since the signal subspace ***E****_s_* is usually substituted for its estimated value ***Ê****_s_*, the unitary matrix **Φ** in Equation (8) becomes:

(24) $$\Phi =\left[\begin{array}{llll} \rho_{1}e^{j\frac{2\pi }{\lambda }d\sin(\theta_{1})} & & & \\ & \rho_{2}e^{j\frac{2\pi }{\lambda }d\sin(\theta_{2})} & & \\ & & \ddots & \\ & & & \rho_{p}e^{j\frac{2\pi }{\lambda }d\sin(\theta_{p})} \end{array}\right]$$

Next, for ease of notation, we define:

(25) $$ϒ=W^{T/2}\left[\begin{array}{llll} E_{s1}^{T} & E_{s2}^{T} & \cdots & E_{sn}^{T} \end{array}\right]$$

and:

(26) $$\Psi =\left[\begin{array}{llll} A_{1}^{T}\Gamma_{1} & \Phi^{{ɛ}_{1}}A_{1}^{T}\Gamma_{2} & \cdots & \Phi^{{ɛ}_{n-1}}A_{1}^{T}\Gamma_{n} \end{array}\right]$$

The expression in Equation (22) is reformulated as:

(27) $$\Omega (\mu )={\left\|ϒ-T_{1}^{T}\Psi \right\|}_{F}^{2}$$

Here, the equations **Φ***^ε_i_T^* = **Φ***^ε_i_^* and **Γ***_i_^T^* = **Γ***_i_* are used.

This is a nonlinear least squares problem for unknown vector **μ** (see [29]). Solving $T_{1}^{T}$ by minimizing the cost function Ω, we can obtain:

(28) $${\hat{T}}_{1}^{T}=ϒ\Psi^{H}{\left(\Psi \Psi^{H}\right)}^{-1}$$

Substituting Equation (28) back to Equation (27) yields:

(29) $$\hat{\mu }=\underset{\mu }{\arg\min}\Omega =\underset{\mu }{\arg\min}{\left\|ϒ\prod_{\Psi }^{⊥}\right\|}_{F}^{2}$$

where:

(30) $$\Pi_{\Psi }^{⊥}=I-\Psi^{H}{\left(\Psi \Psi^{H}\right)}^{-1}\Psi$$

Note that it is reasonable to involve all of the elements of the array to estimate unknown parameters. The limiting case is that there are no overlapping subarrays (*i.e.*, *M* = *mn*). Thus, the matrix **Ψ** has full row rank, except that some elements of the array are not always selected (*i.e.*, *M* < *mn*). Therefore, the matrix (**ΨΨ***^H^*) is nonsingular.

The method considered herein requires a multidimensional search over parameter vector **μ** ∈ 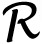
^2(^*^M^*^+^*^p^*^−1)×1^. It is well known that the Gauss–Newton algorithm [29,30] is a classical way to obtain the solution of this problem. Considering the cost function Ω in Equation (29), the unknown parameters can be iteratively obtained by:

(31) $$\mu^{k+1}=\mu^{k}-\xi_{k}H^{-1}Q$$

where **μ***^k^* denotes the estimate at *k*—th iteration, *ξ_k_* is an iterative step length and the gradient vector $Q=\frac{\partial \Omega }{\partial \mu }$ and the Hessian matrix $H=\frac{\partial^{2}\Omega }{\partial \mu \partial \mu^{T}}$ are evaluated at **μ***^k^*.

We define a vector ***r*** formed by columns scanning matrix $ϒ\Pi_{\Psi }^{⊥}$, *i.e.*,

(32) $$r=\text{vec}\{ϒ\prod_{\Psi }^{⊥}\}$$

Then, the cost function Ω becomes:

(33) $$\Omega =r^{H}r$$

In the following, based on the above equation, the expressions for the gradient vector and an approximate Hessian matrix are presented. Firstly, considering the gradient of Ω w.r.t **μ***_i_*, the *i*-th element of the gradient vector ***Q*** is given by:

(34) Qi=∂Ω∂μi=2Re{riHr},

where $r_{i}=\frac{\partial r}{\partial \mu_{i}}$. The *ij*-th component of the Hessian matrix may be expressed as (see [30,31]):

(35) Hij=∂2Ω∂μi∂μj≃2Re{riHrj}

The reasons for this approximation (*i.e.*, ignoring the second derivative of ***r***) can be found in [28,30], and it brings two benefits for the Gauss–Newton iterative algorithm. One is that the approximate Hessian matrix is positive semidefinite and it guarantees −***H***^−1^***Q*** to be a decent direction. The other is that we only need to calculate the first derivative of ***r*** instead of the second one. Furthermore, a useful modification to ill-conditioned cases is to usually use (***H*** + *ζ****I***) in lieu of ***H***, where the diagonal loading factor *ζ* is usually small.

Next, we focus on the first derivative of vector ***r*** in Equation (32). By the aid of the differentiation of the projection matrix [29], ***r****_i_* can be expressed as:

(36) $$r_{i}=-\text{vec}\left\{ϒ\left(\Psi^{†}\Psi_{i}\prod_{\Psi }^{⊥}+{\left(\Psi^{†}\Psi_{i}\prod_{\Psi }^{⊥}\right)}^{H}\right)\right\}$$

where **ψ**^†^ = **ψ***^H^*(**ψψ***^H^*)^−1^ and $\Psi_{i}=\frac{\partial \Psi }{\partial \mu_{i}}$. The expression of **ψ***_i_* can be obtained from Equations (21), (23), (24) and (26).

This is a tiresome calculation process. However, it can be simplified with the observations that $\frac{\partial \Phi^{{ɛ}_{i-1}}A_{1}^{T}\Gamma_{i}}{\partial \gamma_{j}}=0$ if *γ_j_* is not included in matrix **Γ***_i_*, otherwise, $\frac{\partial \Phi^{{ɛ}_{i-1}}A_{1}^{T}\Gamma_{i}}{\partial {\bar{\gamma }}_{j}}=\Phi^{{ɛ}_{i-1}}A_{1}^{T}I_{kk}$ and $\frac{\partial \Phi^{{ɛ}_{i-1}}A_{1}^{T}\Gamma_{i}}{\partial {\bar{\gamma }}_{j}}=j\Phi^{{ɛ}_{i-1}}A_{1}^{T}I_{kk}$, where ***I****_kk_* is a matrix with one in the *kk*–th position and zeros elsewhere, and *γ_j_* is the *k*–th element at the main diagonal of matrix Γ*_j_*. Moreover,

(37) $$\frac{\partial \Phi^{{ɛ}_{i-1}}A_{1}^{T}\Gamma_{i}}{\partial \theta_{j}}=\left(\frac{\partial \Phi^{{ɛ}_{i-1}}}{\partial \theta_{j}}A_{1}^{T}+\Phi^{{ɛ}_{i-1}}\frac{\partial A_{1}^{T}}{\partial \theta_{j}}\right)\Gamma_{i}$$

and:

(38) $$\frac{\partial \Phi^{{ɛ}_{i-1}}A_{1}^{T}\Gamma_{i}}{\partial \rho_{j}}=\frac{\partial \Phi^{{ɛ}_{i-1}}}{\partial \rho_{j}}A_{1}^{T}\Gamma_{i}$$

From the above analysis, the vector ***r****_i_* can be conveniently calculated.

This multidimensional search procedure is briefly described as follows:

1. Estimate the signal subspace ***Ê****_s_*.
2. Initialize vector **μ** Equation (23) utilizing our first method or ESPRIT-like [22].
3. Update **μ** according to Equation (31) fromthe estimates of vector *γ̂* and θ̂*_i_*, *i* = 1, ⋯ , *p*.
4. Calculate matrix **Γ**(*γ̂*) and ***A***(**θ̂**); estimate spatial signature matrix ***V̂*** = **Γ**(*γ̂***)*A***(**θ̂**).
5. Evaluate:
  (39) $$\frac{{\left\|{\hat{V}}_{k+1}-{\hat{V}}_{k}\right\|}_{F}}{{\left\|{\hat{V}}_{k}\right\|}_{F}}$$

where ***V̂****_k_* denotes the estimated spatial signature matrix at the *k*–th step. If it is smaller than a pre-set tolerance, terminate the iterations; if not, repeat Steps 3, 4 and 5.

Remark A: Although our first method and the ESPRIT-like proposed in [22] can only provide rotational values over true DOA **θ** and true gain and phase error matrix **Γ**, it makes no difference to the estimation of spatial signature ***V***. The identifiability of ***V*** from signal subspace ***E****_s_* can be found in [22].

Remark B: It is an intractable problem how to choose weighting matrix ***W*** in Equation (22). Refer to the weighted subspace fitting (WSF) method [31]; we define:

(40) $$W_{\text{opt}}={\left({\hat{\Lambda }}_{s}-{\hat{\sigma }}_{n}^{2}I\right)}^{2}{\hat{\Lambda }}_{s}^{-1}$$

where the diagonal matrix **Λ̂***_s_* contains the so-called signal eigenvalues estimated from the array output sample covariance matrix and ${\hat{\sigma }}_{n}^{2}$ denotes the consistent estimate of noise power. Simulation results using the proposed algorithm show that ***W*** = ***W***_opt_ results in a better performance.

Remark C: The Gauss–Newton algorithm is introduced for solving our nonlinear least squares problem Equation (29). The initial estimate is essential for the global convergence of this iterative method. Fortunately, our first method proposed in Section 3.1 or the ESPRIT-like [22] can provide excellent initial values. Simulation results indicate that only two or three iterative steps are required to achieve a solution close to the global minimum.

Remark D: The computational load of the proposed two methods in this paper is usually dominated by forming the sample covariance matrix ***R̂***, which takes *NM*^2^ flops. The process of obtaining signal subspace ***Ê****_s_* costs 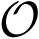
(*M*^2^*p*) flops.

For the first method, the calculation of $\Pi_{E_{x}}^{⊥}$ in Equation (16) requires *p^2^*(*q* − *p*/3) operations if QR-decomposition using Householder transformations is performed, while the calculation of the minimum eigenvalue of $\left(\Pi_{E_{x}}^{⊥}⊙{\left(E_{y}E_{y}^{H}\right)}^{T}\right)$ takes 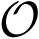
(*q*^2^) flops. Another main computational load is primarily due to the eigendecomposition of **Ξ̂** in Equation (15), and it requires about *p*^2^*q* + 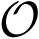
(*p*^3^) flops. The above scheme takes about *q*^2^*p* + 2*p*^2^*q* − *p*^3^/3 + 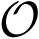
(*p*^3^) flops. When *q* ≫ *p*, our first method requires *NM*^2^ + *q*^2^*p* + 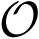
(*M*^2^*p*) flops.

The main computational load of our second method is in the Gauss–Newton iterative process Equation (31) and the first derivative of vector ***r*** Equation (36). The computation of ***r****_i_* needs about *m*^3^*n*^3^ + 3*m*^2^*n*^2^*p* operations, and the inverse matrix of ***H*** in Equation (31) costs approximately 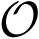
(8(*M* + *p* − 1)^3^) flops. If *M* = *mn* and *M* ≫ *p*, the above two steps take *M*^4^ + 8*M*^3^*p* operations per iteration. Then, our second method requires about *NM*^2^ + 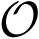
(*M*^4^ + 8*M*^3^*p*) flops.

The main computational complexities of the proposed two algorithms are compared in Table 1 with that of the ESPRIT-like method. In this table, Method 1 denotes the method proposed in Section 3.1, and Method 2 is the approach proposed in Section 3.2. As can be seen from Table 1, the complexity of Method 2 is higher than that of the ESPRIT-like and Method 1. This is because more unknown parameters need to be estimated in Method 2. However, more accurate results can be obtained from this multidimensional search process.

## 4. Experiments

In this section, computer simulations are conducted for evaluating the performance of the proposed algorithms. The approach proposed in Section 3.1 will be referred to as Method 1, while we refer to the method proposed in Section 3.2 as Method 2. In all scenarios, a ULA of 9 elements with half of wavelength element spacing is used. The deterministic gain and phase errors are given by 1, 1.10*e^j^*^10°^, 0.90*e*^−^*^j^*^5°^, 1.25*e^j^*^20°^, 0.80*e*^−^*^j^*^9°^, 0.96*e^j^*^15°^, 1.18*e*^−^*^j^*^23°^, 0.88*e*^−^*^j^*^2°^ and 0.85*e^j^*^4°^. The ULA is divided into 5 subarrays in Method 2, and the *i*-th subarray is selected by matrix:

(41) $$J_{i}=[0_{5\times (i-1)},I_{5\times 5},0_{5\times (5-i)}],i=1,\cdots ,5$$

However, the two subarrays of Method 1 are selected by matrices:

(42) $${\hat{J}}_{1}={\left[\begin{array}{llll} J_{1}^{T} & J_{2}^{T} & J_{3}^{T} & J_{4}^{T} \end{array}\right]}^{T}$$

and:

(43) $${\hat{J}}_{2}={\left[\begin{array}{llll} J_{2}^{T} & J_{3}^{T} & J_{4}^{T} & J_{5}^{T} \end{array}\right]}^{T}$$

Two equal-power signals located at 25° and 30° are considered, and the SNR per element for each source is defined by $\text{SNR}=10\log_{10}(\sigma_{s}^{2}/\sigma_{n}^{2})$, where $\sigma_{s}^{2}$ and $\sigma_{n}^{2}$, respectively, denote the power of the incident signal and that of additive noise at each sensor. RMSE is used as the performance measure and defined by:

(44) $$\text{RMSE}=\frac{1}{K}\sum_{i=1}^{K}\sqrt{{\left\|{\hat{V}}_{i}-V_{i}\right\|}_{F}^{2}}$$

where *K* is the number of trials and ***V̂****_i_* and ***V****_i_* are the estimated spatial signature and the true one at the *i*–th experiment, respectively The result of the ESPRIT-like method in [22] is also included. A total of 200 trials are performed for each simulation scenario. Method 2 terminates after 10 iterations in all simulations.

### Example 1. Value of the Stopping Criterion *Versus* Iteration Number

**Figure 1:**
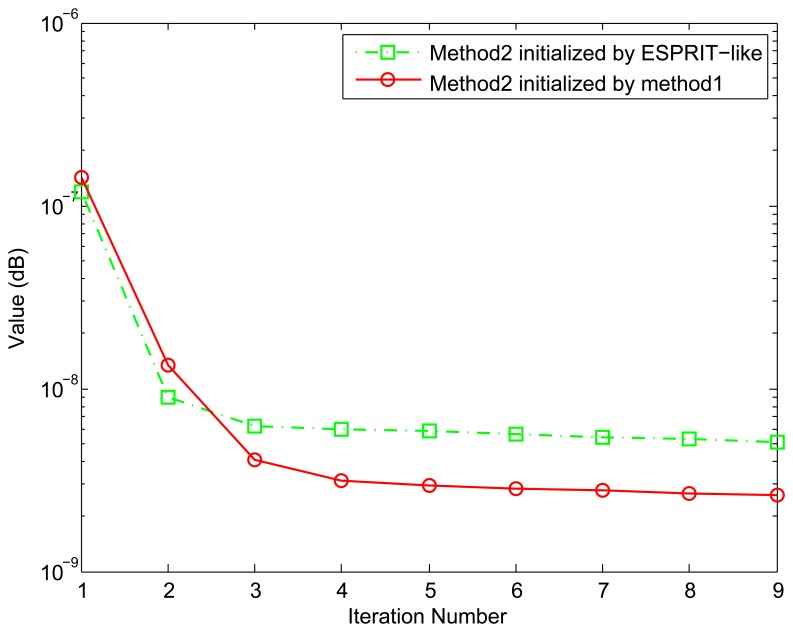
Value of the stopping criterion *versus* iteration number with different initial values. The number of snapshots *N* = 500, and SNR = 15 dB.

Since our proposed Method 2 requires an iterative optimization, it is of interest to evaluate how the estimates improve with each iteration. In this example, Method 2 is initialized separately by Method 1 and ESPRIT-like, and the two incident signals are uncorrelated. Figure 1 shows the value of the stopping criterion Equation (39) as a function of iteration number. It is obvious that all improvement comes in the first two or three iterations. Furthermore, the initial values provided by Method 1 are better than those of ESPRIT-like. This is because more accurate parameter values are estimated by Method 1, and we will show it in the next example.

### Example 2. Performance *Versus* SNR

In the second example, the proposed two methods and the ESPRIT-like in [22] are compared against the SNR. We assume that the incident two signals are uncorrelated, and the number of snapshots is *N* = 500. The SNR is varied from −10 to 10 dB. The weighting matrix ***W*** = ***W***_opt_ in Method 2 is calculated by Equation (40). Moreover, the initial value is given separately by ESPRIT-like and the Method 1.

**Figure 2:**
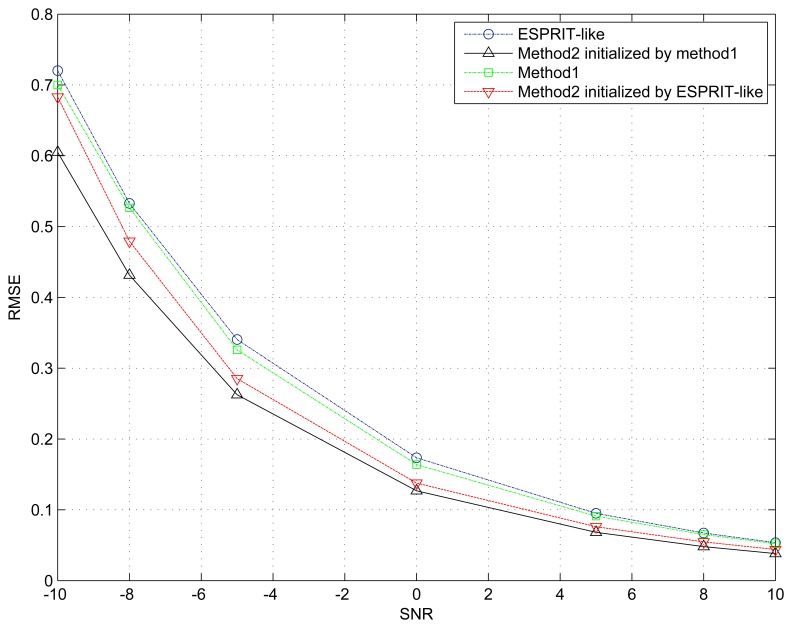
RMSE of the spatial signature matrix ***V*** estimation *versus* SNR. The number of snapshots *N* = 500.

The simulation result in Figure 2 shows that our proposed two algorithms perform better than ESPRIT-like, even at low SNR. This means that the maximum overlapping subarray is not the optimal choice in the presence of unknown sensor gain and phase response. In other words, fully exploiting invariance in the array should be taken into account to construct an estimator. In addition, the behavior of the multidimensional search procedure (Method 2) has been improved when the initial parameter values are provided by our Method 1. Simulation results also indicate that our method only requires 2 to 4 Gauss–Newton iterations, and the main performance improvement comes from the first two iterations.

### Example 3. Performance *Versus* Different ***W***

**Figure 3:**
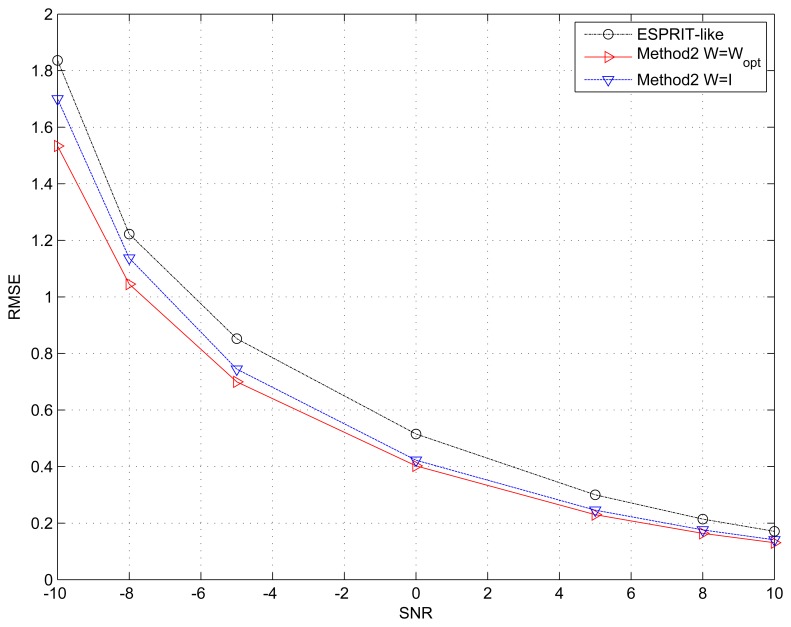
RMSE of the spatial signature matrix ***V*** estimation *versus* SNR with different ***W***. The number of snapshots *N* = 500.

In the third example, we give the simulation results of the multidimensional search procedure (Method 2) with different weighting matrix ***W*** = ***W***_opt_, and ***W***= ***I***. The two sources are also uncorrelated, and the snapshots are fixed at *N* = 500. Method 2 is only initialized by the ESPRIT-like method. The advantage of using the weighting matrix ***W***_opt_ is clearly demonstrated by Figure 3. The introduction of weighting matrix ***W*** can improve the performance of the proposed algorithm especially under low SNR. Meanwhile, this example also shows that an algorithm that fully exploits the physical structure of the array can give better performance.

### Example 4. Performance *Versus* Correlation between Signals

**Figure 4:**
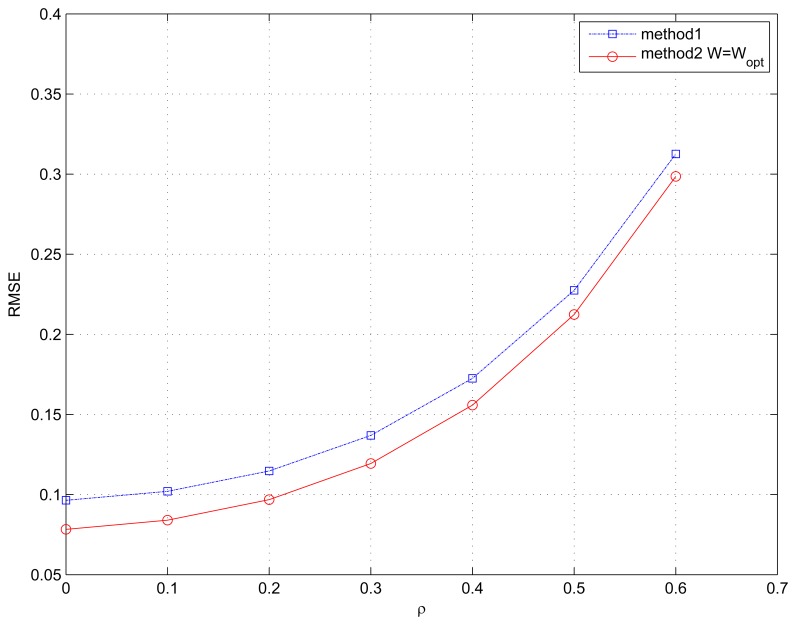
RMSE of the spatial signature matrix ***V*** estimation *versus* correlation factor ρ. The number of snapshots *N* = 1000 and SNR = 15 dB.

In the last one, we test the estimation performance in terms of the correlation between the two incident signals, where the correlation factor is denoted by ρ. The number of snapshots is *N* = 1000 and SNR is fixed at 15 dB. The generations of the two correlated signals can be found in [32]. The inputs of the multidimensional search process are given by Method 1 and the weighting matrix ***W*** = ***W***_opt_. As depicted in Figure 4, our proposed two algorithms perform excellently for correlated signals. However, because the weighting matrix ***W*** is used in Method 2, the multidimensional search algorithm is superior to Method 1.

## 5. Conclusions

The ESPRIT-like method proposed in [22] just makes use of the maximum overlapping subarray of ULA with unknown gain and phase errors to formulate an estimator. However, there exist many invariances in this kind of ULA. Thus, we firstly presented a simple eigenstructure-based algorithm for spatial signature estimation. One element of the ULA can be selected several times in one subarray, while the same element can only be used once in one subarray of the ESPRIT-like method. Another multidimensional search algorithm was also proposed. This method fully excavates the multiple invariances of the ULA and is implemented with the help of the Gauss–Newton iterative algorithm. Because excellent initial parameter values can be provided by the ESPRIT-like or our first method, this algorithm can converge rapidly to an appropriate solution. On the other hand, the introduction of a weighting matrix ***W*** further improves the performance of the multidimensional search algorithm. However, how to select subarrays from ULA, making the algorithm lead to optimal parameter estimation, is not addressed in this paper. Some pragmatic discussion about this question can be found in [27].

## Figures and Tables

**Table 1:** Computational complexity of the ESPRIT-like and the proposed two techniques.

**Algorithm**	**Computational Complexity**
ESPRIT-like	*NM*^2^ + 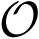 (2*M*^2^*p*)
Method 1	*NM*^2^ + *q*^2^*p* + 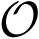 (*M*^2^*p*)
Method 2	*NM*^2^ + 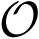 (*M*^4^ + 8*M*^3^*p*)
